# Intrathecal Isoniazid and Dexamethasone Therapy May Improve Outcomes in Patients With Tuberculous Meningitis: A 2-Center Retrospective Cohort Study

**DOI:** 10.1093/cid/ciag106

**Published:** 2026-02-18

**Authors:** Rui Chen, Yaojie Shen, Huarui Liu, Meiji Wang, Zhentao Fei, Lu Xia, Dan Ye, Yang Ren, Xiaoqing Ma, Yang Yang, Meijun Liu, Xuhui Liu, Yuanyuan Chen, Feng Li

**Affiliations:** Shanghai Institute of Infectious Disease and Biosecurity, Fudan University, Shanghai, China; Department of Pulmonary and Critical Care Medicine, Shanghai Public Health Clinical Center, Fudan University, Shanghai, China; Department of Tuberculosis, Hangzhou Red Cross Hospital, Hangzhou, Zhejiang, China; Department of Tuberculosis, Shanghai Public Health Clinical Center, Fudan University, Shanghai, China; Institute for AIDS/STD Prevention and Control, Yunnan Center for Disease Control and Prevention, Kunming, Yunnan, China; Department of Tuberculosis, Shanghai Public Health Clinical Center, Fudan University, Shanghai, China; Department of Tuberculosis, Shanghai Public Health Clinical Center, Fudan University, Shanghai, China; Tuberculosis Research Center, Shanghai Public Health Clinical Center, Fudan University, Shanghai, China; Department of Tuberculosis, Shanghai Public Health Clinical Center, Fudan University, Shanghai, China; Department of Pulmonary and Critical Care Medicine, Shanghai Public Health Clinical Center, Fudan University, Shanghai, China; Department of Tuberculosis, Hangzhou Red Cross Hospital, Hangzhou, Zhejiang, China; Department of Tuberculosis, Shanghai Public Health Clinical Center, Fudan University, Shanghai, China; Shanghai Institute of Infectious Disease and Biosecurity, Fudan University, Shanghai, China; Department of Pulmonary and Critical Care Medicine, Shanghai Public Health Clinical Center, Fudan University, Shanghai, China; Department of Tuberculosis, Shanghai Public Health Clinical Center, Fudan University, Shanghai, China; Tuberculosis Research Center, Shanghai Public Health Clinical Center, Fudan University, Shanghai, China; Department of Tuberculosis, Hangzhou Red Cross Hospital, Hangzhou, Zhejiang, China; Shanghai Institute of Infectious Disease and Biosecurity, Fudan University, Shanghai, China; Department of Pulmonary and Critical Care Medicine, Shanghai Public Health Clinical Center, Fudan University, Shanghai, China; Tuberculosis Research Center, Shanghai Public Health Clinical Center, Fudan University, Shanghai, China

**Keywords:** intrathecal treatment, tuberculous meningitis, modified Rankin Scale score, propensity score matching

## Abstract

**Background:**

This study aims to evaluate the effect of intrathecal (IT) administration of antituberculosis agents on functional outcomes in patients with tuberculous meningitis (TBM) and to explore its potential as a strategy to overcome the blood–brain barrier.

**Methods:**

We retrospectively analyzed TBM patients admitted between 2012 and 2023 at Hangzhou Red Cross Hospital and Shanghai Public Health Clinical Center. Clinical data were collected during hospitalization, and functional outcomes were assessed at 3–6 months after discharge using the modified Rankin Scale (mRS). Propensity score matching (PSM) was applied to balance baseline characteristics between patients who received IT therapy and those who did not. Treatment effects were compared using the Mann–Whitney U test.

**Results:**

A total of 533 patients with TBM were included. After PSM, 72 patients receiving IT therapy were compared with 121 matched controls. Intrathecal therapy was associated with significantly lower mRS scores at discharge compared with controls (*P* = .0012), suggesting that it may improve functional outcomes.

**Conclusions:**

Intrathecal administration of isoniazid and dexamethasone may improve functional prognosis in TBM patients. These findings highlight IT therapy as a promising approach to circumvent the blood–brain barrier and warrant validation in multicenter randomized controlled trials.

## Teaser

In a dual-center retrospective study of 533 adults with tuberculous meningitis, adjunctive intrathecal isoniazid and dexamethasone improved functional outcomes compared to systemic therapy alone, with consistent results after propensity score matching. Prospective randomized trials are needed to confirm these findings.

Tuberculous meningitis (TBM) represents the most devastating form of extrapulmonary tuberculosis. Although its incidence is relatively low, TBM is associated with exceptionally high rates of disability and mortality, making it a critical global public health challenge [1, 2]. Epidemiological estimates indicate that TBM accounts for 1%–5% of all tuberculosis cases and 13.9% of meningitis cases worldwide, causing approximately 78 200 adult deaths annually—up to 35% of which occur among individuals with human immunodeficiency virus (HIV)/AIDS [3]. Delayed diagnosis and treatment can drive case fatality rates beyond 50% and frequently result in severe neurological sequelae [4]. Pharmacokinetic studies further demonstrate striking heterogeneity in the central nervous system (CNS) penetration of antituberculosis agents. For example, rifampicin (RIF) and ethambutol (EMB) often fail to reach bactericidal concentrations in cerebrospinal fluid (CSF), thereby limiting therapeutic efficacy [5, 6].

Intrathecal (IT) administration has emerged as a potential therapeutic strategy for TBM [7]. By enabling sustained and localized drug delivery into the CSF, this approach markedly increases local drug concentrations while reducing systemic exposure and associated toxicities [8]. Since the introduction of IT or intraventricular isoniazid (INH) in 1955 and RIF in 1976, this route has demonstrated the capacity to directly target the CNS, minimize systemic toxicity, and improve clinical outcomes, with both experimental and theoretical evidence supporting its pharmacological rationale [9–12]. Preclinical studies and case reports have further shown that drugs such as INH and amikacin (AMK), when administered intrathecally, can achieve high CSF concentrations and exert potent local effects [13, 14]. Nevertheless, the clinical utility of IT therapy in TBM remains controversial. Concerns center on potential neurotoxicity, procedure-related complications, and the absence of robust evidence from large-scale randomized controlled trials [15]. Consequently, current international guidelines have not endorsed IT administration as part of routine care, restricting its use to adjunctive therapy in selected severe or refractory cases [16, 17].

Given the uncertainty surrounding the precise role of IT in TBM, there is an urgent need for clinical studies to rigorously evaluate its efficacy and safety. In this study, we conducted a retrospective cohort analysis employing propensity score matching (PSM) to compare outcomes between TBM patients who received IT therapy and those who did not. We aimed to determine the impact of IT administration on clinical prognosis and provide evidence to inform its potential integration into clinical practice.

## METHODS

### Study Design and Patients

This was a retrospective cohort study based on clinical records, designed to evaluate the impact of IT therapy on clinical outcomes in patients with TBM. Eligible participants were adults aged ≥18 years who were diagnosed with TBM. We extracted clinical data from two tertiary Grade A infectious diseases hospitals in China—Hangzhou Red Cross Hospital and Shanghai Public Health Clinical Center—covering an 11-year period (November 2012–November 2023). Both institutions are designated tuberculosis treatment centers that primarily manage pulmonary tuberculosis while also providing care for extrapulmonary disease, including TBM; neither functions as a dedicated tertiary referral center. The study protocol was approved by the Ethics Committee of the Shanghai Public Health Clinical Center (Approval No. 2024-S002-01) and conducted in accordance with the principles of the Declaration of Helsinki. Given the retrospective design, the requirement for written informed consent was waived.

We included TBM patients aged ≥18 years, with case definitions based on the standardized diagnostic criteria proposed by Marais et al (2010). Patients were categorized as follows: (1) definite TBM, microbiological confirmation of *Mycobacterium tuberculosis* in CSF or meningeal tissue; (2) probable TBM, absence of direct microbiological evidence, but a diagnostic score above the recommended threshold, derived from clinical features, CSF findings, neuroimaging characteristics, and/or evidence of prior tuberculosis; and (3) possible TBM, lower diagnostic scores, yet consistent clinical manifestations without a more plausible alternative diagnosis. Patients meeting any of the above criteria and with available outpatient follow-up data were included in the analysis [18]. Given the retrospective design and China's centralized care pathway for HIV infection—whereby HIV-positive individuals are routinely managed at designated AIDS treatment centers—we excluded patients with HIV to ensure data completeness and cohort comparability.

Patients were divided into a control group and a treatment group. The control group received standard antituberculosis therapy in accordance with the World Health Organization (WHO) guidelines: an intensive phase consisting of daily INH, RIF, PZA, and EMB for 2 months, followed by a continuation phase of daily INH and RIF for 10 months. In addition to this standard regimen, patients in the treatment group received IT. Intrathecal administration was performed via lumbar puncture at the L3/4 or L4/5 interspace, with drugs delivered into the subarachnoid space. Following injection, patients were required to remain supine and at rest for 4–6 hours to minimize complications and facilitate drug distribution. The IT regimen consisted of a weekly injection of a mixture containing 50 mg INH and 2.5 mg dexamethasone. The duration and total number of IT administrations were determined by the treating physician, based on the severity of disease and the overall clinical assessment. Across both centers, the IT regimen and administration procedure were similar in routine practice. No protocol-mandated stopping criteria were applied; initiation, discontinuation, and the total number of injections were determined by the treating physician based on overall clinical response, CSF parameters, and tolerability (range, 2–12; mean, 5). Cerebrospinal fluid or plasma isoniazid concentrations were not routinely monitored and were therefore unavailable for analysis.

### Data Collection

Clinical data were systematically extracted from medical records by trained investigators. Information collected included demographic characteristics, clinical presentations, treatment details (including IT administration), neuroimaging findings, and functional outcomes. Additional follow-up data were obtained 3–6 months after discharge through outpatient visits. For patients who had died or could not be contacted directly, interviews were conducted with first-degree relatives. Only patients with complete follow-up information were included in the final analysis. Procedure-related adverse events potentially attributable to lumbar puncture or IT administration were identified through structured review of procedure notes, daily progress notes, nursing documentation, and discharge summaries. Disease severity was classified according to the British Medical Research Council (BMRC) staging system, categorizing patients into stages I, II, or III. To minimize confounding, PSM was applied, incorporating variables, such as age, sex, number and duration of hospitalizations, comorbidities, limb weakness, and altered consciousness. These factors were selected both as essential demographic and clinical characteristics and because previous studies have demonstrated their strong association with prognosis [19, 20].

### Functional Outcomes

In the propensity score–matched cohort, we compared functional outcomes between treatment groups based on outpatient follow-up records. The primary outcome measure was the modified Rankin Scale (mRS) score at 3–6 months after hospital discharge. The mRS ranges from 0 to 6, with scores of 0–2 defined as a favorable outcome and scores of 3–6 as an unfavorable outcome [21].

### Statistical Analysis

Data are presented as mean ± SD or as percentages, depending on variable type. Given the nonrandomized design of this study and the potential for baseline imbalances, PSM was applied to achieve covariate balance. Specifically, nearest-neighbor matching was performed at a 1:2 ratio with a caliper width of 0.2. Between-group comparisons were conducted using Student *t* test for continuous variables and the chi-square test for categorical variables. All statistical tests were 2-sided, and a *P* value of <.05 was considered statistically significant. Statistical analyses were performed using R software (version 4.5.0; R Foundation for Statistical Computing, Vienna, Austria). Covariate balance after matching was assessed by inspection of the common support region of propensity scores and standardized mean differences (SMDs) [22]. Patients without sufficient propensity score overlap (outside the region of common support) or without an eligible match within the prespecified caliper were excluded from the matched analysis.

## RESULTS

### Patient Characteristics

A total of 581 patients met the diagnostic criteria for TBM, of whom 48 were excluded due to incomplete clinical records. Ultimately, 533 patients were included in the analysis. Among them, 332 (62.3%) were male and 201 (37.7%) were female, with a median age of 48 years (interquartile range [IQR], 30–61 years). Based on diagnostic classification, 266 (49.9%) were definite TBM, 100 (18.8%) probable TBM, 51 (9.6%) possible TBM, and 116 (21.8%) unclassified TBM. According to the BMRC staging system, 307 (57.6%) patients were classified as stage I, 166 (31.1%) as stage II, and 60 (11.3%) as stage III.

Among IT-treated patients (n = 100), the number of injections ranged from 2 to 12 (mean, 5). Overall, 7 (7.0%) experienced procedure-related adverse events, including local discomfort (n = 5), minor bleeding at the puncture site (n = 1), and transient neurological symptoms (n = 1), manifested as urinary retention and mild lower limb pain. No chemical arachnoiditis was observed. All adverse events resolved spontaneously within 1–3 days, and no delayed neurological complications attributable to IT therapy were documented during the 3–6-month follow-up.

### Propensity Score Matched Analysis

**Figure 1.**
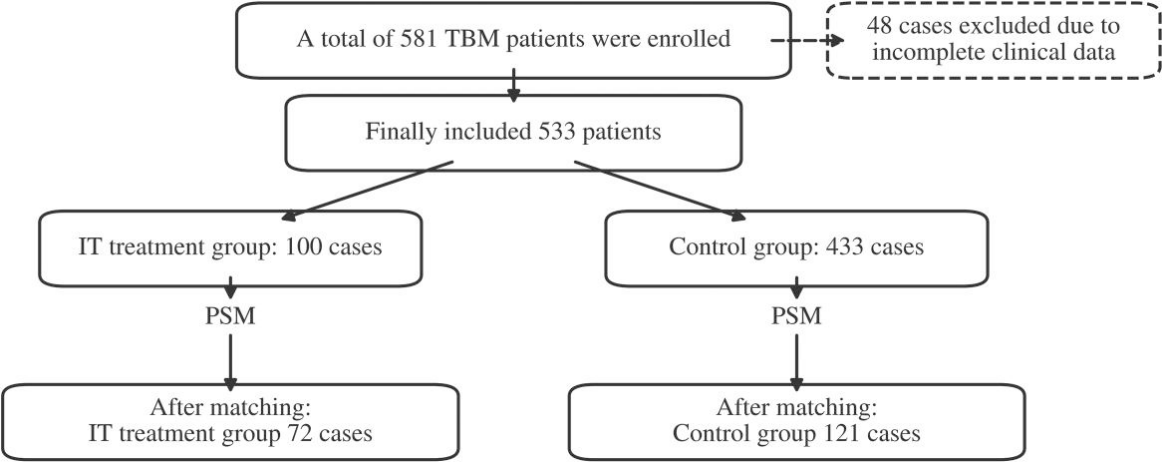
Enrollment flow of patients with tuberculous meningitis (TBM). The diagram illustrates the screening, exclusion, and final inclusion of eligible participants.

Before PSM, a total of 533 TBM patients were included, comprising 433 in the control group and 100 in the IT treatment group (Figure 1). No significant differences were observed between the 2 groups with respect to age, sex, length of hospitalization, Glasgow Coma Scale (GCS) score, CSF appearance, adenosine deaminase (ADA) level, or intracranial pressure (*P* > .05 for all). However, the IT group had a significantly higher median number of hospitalizations compared with the control group (2 vs 1, *P* < .001) and a markedly greater proportion of smokers (33.0% vs 14.1%, *P* < .001). Further comparison of admission-related disease severity revealed that the IT group exhibited more severe clinical presentation at baseline. Specifically, higher rates of limb weakness (*P* = .029), elevated positivity for the Pandy test (*P* < .001), greater CSF white blood cell counts (*P* < .001), increased lactate dehydrogenase (LDH) levels (*P* = .014), and lower CSF chloride concentrations (*P* = .022) were observed compared with the control group. Collectively, these findings suggest that patients selected for IT therapy tended to present with more advanced disease and heightened inflammatory responses at admission (Table 1).

**Table 1.** Baseline Clinical Characteristics of Tuberculous Meningitis (TBM) Patients Prior to Propensity Score Matching (PSM)

Variable	Overall (n = 533)	Control Group (n = 433)	Intrathecal Therapy Group (n = 100)	*P* Value
TBM diagnostic categories, n (%)	…	…	…	<.001
Definite	266 (49.9%)	207 (47.8%)	59 (59.0%)	…
Probable	100 (18.8%)	73 (16.9%)	27 (27.0%)	…
Possible	51 (9.6%)	49 (11.3%)	2 (2.0%)	…
Unclassified	116 (21.8%)	104 (24.0%)	12 (12.0%)	…
Age (years)	48 [30, 61]	48 [30, 61]	49.5 [28, 60]	.879
Sex, n (%)	…	…	…	.386
Male	332 (62.3)	274 (63.3)	58 (58.0)	…
Female	201 (37.7)	159 (36.7)	42 (42.0)	…
Number of hospitalizations	1 [1, 1]	1 [1, 1]	2 [1, 3]	<.001
Length of hospital stay (days)	29 [17, 45]	29 [15, 46]	30 [22.75, 43.25]	.208
Smoking history, n (%)	94 (17.6)	61 (14.1)	33 (33.0)	<.001
Comorbidities, n (%)	…	…	…	…
Hypertension	79 (14.8)	70 (16.2)	9 (9.0)	.097
Diabetes mellitus	55 (10.3)	49 (11.3)	6 (6.0)	.164
Clinical signs and laboratory indices	…	…	…	…
Fever	428 (80.3)	341 (78.8)	87 (87.0)	.084
Headache	356 (66.8)	281 (64.9)	75 (75.0)	.069
Limb weakness	70 (13.1)	64 (14.8)	6 (6.0)	.029
Glasgow Coma Scale (GCS)	15 [13, 15]	15 [13, 15]	15 [13, 15]	.366
mMRC score	1 [1, 2]	1 [1, 2]	1 [1, 2]	.047
White blood cell count (×10^9^/L)	6.67 [4.86, 8.85]	6.67 [4.86, 9.00]	6.65 [5.08, 8.07]	.511
Abnormal CSF appearance, n (%)	22 (4.1)	15 (3.5)	7 (7.0)	.160
Pandy test positive, n (%)	466 (87.4)	367 (84.8)	99 (99.0)	<.001
CSF white cell count (cells/μL)	80 [20, 200]	60 [12, 172]	155 [80, 287]	<.001
Lactate dehydrogenase (LDH, U/L)	51 [31, 85]	48 [29, 82]	57.5 [38, 89.25]	.014
CSF chloride (mmol/L)	115 [109, 120.3]	116 [109.1, 121]	113.15 [107.97, 119]	.022
Adenosine deaminase (ADA, U/L)	5 [2, 9.2]	5 [1, 10]	5.15 [4, 8]	.095
Intracranial pressure (mmH_2_O)	180 [120, 260]	170 [120, 250]	200 [137.5, 292.5]	.195

Data are presented as median [IQR] or n (%), unless otherwise indicated.

After PSM, a total of 193 matched cases were obtained, including 121 in the control group and 72 in the IT treatment group. The matching procedure substantially reduced baseline imbalances, with no statistically significant differences observed between the 2 groups in most clinical characteristics, including age, sex, number and duration of hospitalizations, comorbidities, vital signs, and laboratory parameters (*P* > .05 for all). This indicated that the baseline features of the 2 groups were largely comparable after matching. Notably, a residual difference remained in CSF white blood cell counts, which were significantly higher in the IT group compared with the control group (150 [60–280] versus 96.5 [20.5–220], *P* = .046) (Table 2). Of the 100 IT-treated patients, 72 were successfully matched, whereas 28 could not be matched within the prespecified caliper (ie, outside the region of common support) and were therefore excluded from the matched analysis.

**Table 2.** Baseline Clinical Characteristics of Tuberculous Meningitis (TBM) Patients After Propensity Score Matching (PSM)

Variable	Overall (n = 193)	Control Group (n = 121)	Intrathecal Therapy Group (n = 72)	*P* Value
TBM diagnostic categories, n (%)	…	…	…	.187
Definite	114 (59.1%)	74 (61.2%)	40 (55.6%)	…
Probable	41 (21.2%)	20 (16.5%)	21 (29.2%)	…
Possible	13 (6.7%)	9 (7.4%)	4 (5.6%)	…
Unclassified	25 (13.0%)	18 (14.9%)	7 (9.7%)	…
Age (years)	50 [30, 63]	49 [31, 63]	51.5 [29.5, 61.25]	.920
Sex, n (%)	…	…	…	1.000
Male	108 (56.0)	68 (56.2)	40 (55.6)	…
Female	85 (44.0)	53 (43.8)	32 (44.4)	…
Number of hospitalizations	1 [1, 2]	1 [1, 2]	1 [1, 2]	.620
Length of hospital stay (days)	28 [19, 40]	27 [19, 42]	28 [19.5, 36.5]	.750
Smoking history, n (%)	50 (25.9)	29 (24.0)	21 (29.2)	.530
Comorbidities, n (%)	…	…	…	…
Hypertension	26 (13.5)	18 (14.9)	8 (11.1)	.601
Diabetes mellitus	19 (9.8)	13 (10.7)	6 (8.3)	.769
Clinical signs and laboratory indices	…	…	…	…
Fever	162 (83.9)	100 (82.6)	62 (86.1)	.666
Headache	138 (71.5)	85 (70.2)	53 (73.6)	.737
Limb weakness	15 (7.8)	9 (7.4)	6 (8.3)	1.000
Glasgow Coma Scale (GCS)	15 [14, 15]	15 [14, 15]	15 [13.75, 15]	.075
mMRC score	1 [1, 2]	1 [1, 2]	1 [1, 2]	.845
White blood cell count (×10^9^/L)	6.48 [4.73, 8.3]	6.32 [4.73, 8.2]	6.6 [5.08, 8.35]	.624
Abnormal CSF appearance, n (%)	10 (5.2)	6 (5.0)	4 (5.6)	1.000
Pandy test positive, n (%)	192 (99.5)	121 (100.0)	71 (98.6)	.792
CSF white cell count (cells/μL)	110 [40, 255]	96.5 [20.5, 220]	150 [60, 280]	.046
Lactate dehydrogenase (LDH, U/L)	52 [34, 85]	49 [32, 82]	53.5 [35.75, 85]	.379
CSF chloride (mmol/L)	114.7 [109.3, 118.9]	115 [109, 119]	114 [110, 118.58]	.766
Adenosine deaminase (ADA, U/L)	5 [3, 8.4]	5.1 [2.55, 9.0]	5 [4, 8]	.583
Intracranial pressure (mmH_2_O)	200 [120, 280]	190 [120, 280]	200 [120, 262.5]	.851

Data are presented as median [IQR] or n (%), unless otherwise indicated.

### Outcomes

Functional outcome was assessed using the mRS (0–6), where lower scores indicate better functional status [12–14]. Before PSM, patients in the IT group had a median mRS score of 0 (IQR, 0–1), significantly lower than that of the control group (median, 1; IQR, 0–3; *P* < .001). The corresponding odds ratio (OR) was 0.27 (95% CI, .17–.43), indicating that IT therapy was associated with more favorable functional outcomes. After PSM to balance baseline characteristics, this difference remained significant: The median mRS score in the IT group was 0 (IQR, 0–1) versus 1 (IQR, 0–1) in the control group (*P* = .0012), with an OR of 0.37 (95% CI, .20–.68) (Table 3). Taken together, both pre- and postmatching analyses consistently demonstrate that IT therapy is linked to superior functional prognosis in patients with TBM.

**Table 3.** Association Between Intrathecal Therapy and Clinical Outcomes in Patients With Tuberculous Meningitis (TBM)

Clinical Outcome Measure	Overall	Control Group	Intrathecal Therapy Group	*P* Value	OR (95% CI)
Before matching (n = 533)	n = 533	n = 433	n = 100	…	…
Modified Rankin Scale (mRS)	1 [0, 3]	1 [0, 3]	0 [0, 1]	<.001	.27 (.17, .43)
After matching (n = 193)	n = 193	n = 121	n = 72	…	…
Modified Rankin Scale (mRS)	1 [0, 1]	1 [0, 1]	0 [0, 1]	.0012	.37 (.20, .68)

**Figure 2.**
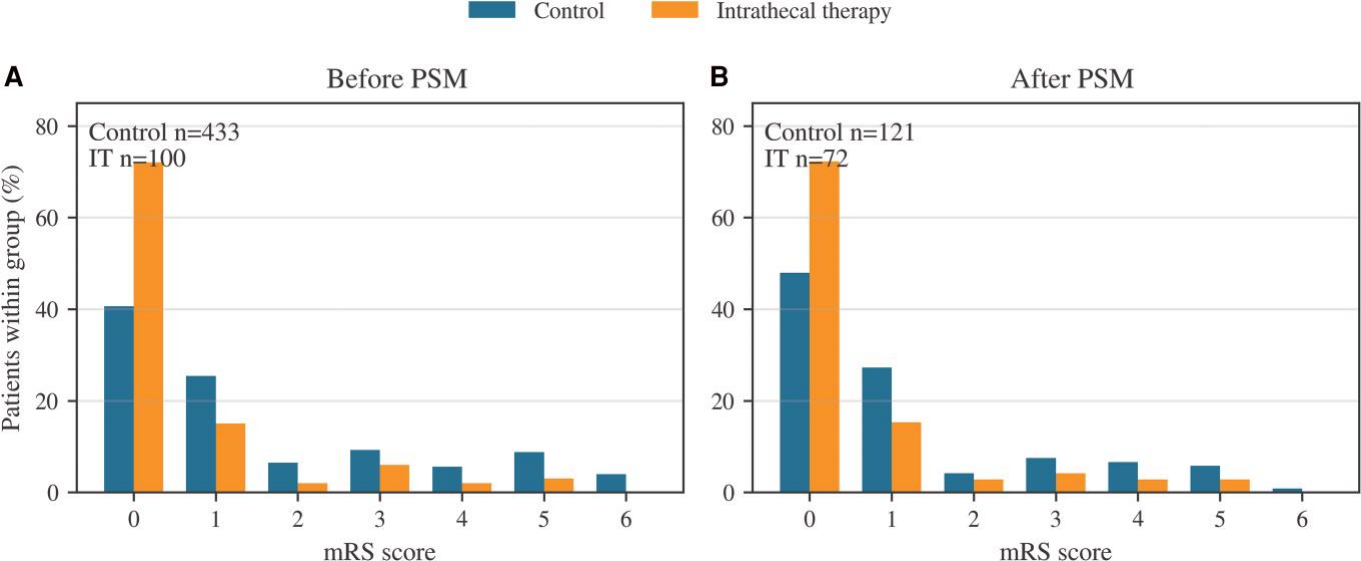
Within-group percentage distribution of modified Rankin Scale (mRS) scores before and after propensity score matching (PSM).

Figure 2 shows the within-group percentage distribution of mRS scores in the IT therapy and control groups before and after PSM.

## DISCUSSION

Tuberculous meningitis represents one of the most severe manifestations of tuberculosis, with high rates of disability and mortality [23]. In this 2-center retrospective cohort, adjunctive IT therapy was associated with better functional outcomes compared with systemic antituberculosis treatment alone. This association was consistent in both the overall cohort and the propensity score–matched analysis, supporting the robustness of the observed benefit after balancing measured baseline characteristics.

Overall, the suboptimal treatment outcomes seen in TBM are largely attributable to 2 major challenges: (1) Systemically administered antituberculosis drugs poorly penetrate the BBB, resulting in insufficient drug concentrations in the CSF and (ii) systemic dose escalation, while capable of increasing drug exposure within the CNS, is frequently constrained by systemic toxicities, particularly hepatotoxicity and other adverse effects. The principal advantage of IT therapy lies in its ability to bypass the blood–brain barrier (BBB) and deliver drugs directly into the CNS, thereby markedly increasing CSF drug concentrations while minimizing systemic toxicities [24–26].

Our findings are consistent with the limited but suggestive body of prior evidence regarding IT therapy in TBM. Early reports (eg, Swift and colleagues) described favorable microbiological and clinical responses following IT INH in small cohorts [27]. A subsequent meta-analysis evaluating IT dexamethasone combined with INH suggested improved overall efficacy and reduced risk of poor outcomes without a clear increase in adverse events [28–30]. More recent observational studies have also reported improved long-term outcomes when IT therapy was added to systemic regimens and have proposed more individualized strategies integrating optimized systemic therapy with innovative IT delivery and multimodal management. By leveraging PSM within a dual-center real-world cohort, our study demonstrates that IT therapy, when combined with systemic antituberculosis treatment, is associated with a more pronounced improvement in prognosis compared with systemic therapy alone.

Pharmacokinetic considerations further support a potential role for IT therapy [31–35]. High-dose systemic INH has been associated with improved survival in TBM, but dose escalation is often constrained by systemic toxicity [36]. Intraventricular isoniazid exposure is strongly influenced by NAT2 acetylator status, and rapid acetylators—reported to be relatively common in Chinese populations—may have lower plasma concentrations and higher risk of treatment failure under standard dosing [28].

Intrathecal delivery can substantially increase CSF INH levels, which may be particularly advantageous when systemic exposure is limited by rapid metabolism or dose-limiting toxicity. These pharmacological factors provide a plausible mechanistic explanation for the improved functional outcomes observed with adjunctive IT therapy in our cohort [37–40].

Safety is a key concern when considering IT administration. While prior case reports have described neurological events (eg, seizures, optic atrophy, hemiplegia, and mild cerebral herniation that is typically reversible) and transient jaundice after IT administration of RIF or isoniazid [41–43], in our cohort, adverse events were generally mild and self-limited, resolving spontaneously within 1–3 days, and no delayed neurological complications attributable to IT therapy were documented during the 3–6-month follow-up. These findings suggest that, when performed with appropriate technique and monitoring, IT therapy may be feasible and tolerable; however, standardized protocols and careful patient selection remain essential [44–46].

However, several limitations should be acknowledged: First, the absence of blinding may introduce potential bias in the analysis process. Second, follow-up information for some patients was obtained via telephone recalls from family members or primary clinicians, which may lead to recall bias. Third, although the proportion of “possible TBM” cases was small, including these patients still introduces a risk of misclassification. Fourth, heterogeneity in treatment protocols, including concomitant medication regimens, across different centers could influence patient outcomes. Finally, other unaccounted-for confounding factors might impact the analysis of the study results. Therefore, the conclusions of this study require further validation through large-scale, prospective, multicenter trials to provide more robust evidence for the clinical management of TBM.

In summary, compared to standard therapy alone, patients who received combination IT treatment demonstrated superior functional outcomes at follow-up, suggesting that IT therapy may represent an effective strategy to overcome the limitations imposed by the BBB and improve the prognosis of TBM patients [47–50]. Prospective multicenter studies—ideally randomized controlled trials—are warranted to confirm efficacy, define optimal candidates and regimens, and further characterize short- and long-term safety.
